# The role of meniscal repair for prevention of early onset of osteoarthritis

**DOI:** 10.1186/s40634-018-0122-z

**Published:** 2018-04-02

**Authors:** Johannes Weber, Matthias Koch, Peter Angele, Johannes Zellner

**Affiliations:** 1 Department of Trauma Surgery, University Hospital of Regensburg, Franz-Josef-Strauss-Allee 11, 93053 Regensburg, Germany; 2 sporthopaedicum Regensburg/Straubing, Hildegard von Bingen Strasse 1, 93053 Regensburg, Germany

**Keywords:** Meniscus, Meniscus repair, Prevention, Osteoarthritis of the knee, Review

## Abstract

**Background:**

The meniscus plays an important role in the integrity of the knee joint. Therefore, meniscus tissue preserving techniques for the therapy of meniscus injuries seem to be reasonable. One of the important questions is whether meniscal repair can prevent the knee joint from early onset of osteoarthritis.

**Methods:**

According to the review of the current literature, the principles of a successful meniscal repair are explained and the functional outcome and its impact on the prevention of osteoarthritis are analyzed in this article.

**Results:**

Current data show a positive impact of a successful meniscus repair on the functional outcome in long-term. By this a protective effect on the development of osteoarthritis via the repair of meniscus lesions to restore the meniscus integrity can be confirmed. However, higher rates of re-operations in context to meniscus suturing have to be considered.

**Conclusion:**

Due to the improved functional outcomes as well as preventive effect on the development of osteoarthritis within the knee joint in long-term, it is of importance to preserve as much meniscus tissue as possible in meniscus therapy. Patients previously have to be informed about the higher revision rate in context to meniscus suturing.

## Review

### Introduction

The meniscus plays a decisive role for the integrity of the knee joint. It includes shock absorption and transmission, but also joint stabilization and proprioception as well as lubrication and nutrition of the articular cartilage (Makris et al., 2011). Intervention on the meniscus, in particular partial meniscectomies, are one of the most frequently performed operative orthopaedic procedures and underline the impact of this subject. Biomechanical studies have shown that a loss of the meniscus integrity leads to a marked change in kinematics and load distribution in the knee joint. The pressure on the surrounding native articular cartilage subsequently increases. Even a resection of 15–34% of the meniscus tissue additionally enhances the load on the hyaline cartilage up to 350% (Radin et al., 1984).

In accordance to that, gonarthrosis as a resulting effect of meniscectomy has already been first described a long time ago (Fairbank, 1948) and according to current literature partial meniscectomy is also well known to predispose for the development of gonarthrosis (McDermott & Amis, 2006; Petty & Lubowitz, 2011).

Following criterias are defined as risk factors for the development of degenerative changes in context to meniscus injuries (according to Mordecai) (Mordecai et al., 2014):

- partial resection of the lateral meniscus
- resection of larger portions of meniscus tissue
- radial tears reducing or canceling the meniscus ring tension (functional meniscectomy)
- pre-existing cartilage lesions
- persisting ligamentary joint instability
- axial deviation (varus-medial, valgus-lateral)
- obesity
- age > 40 years
- low activity level

According to the increasing knowledge concerning the biology and function of the meniscus there is a consensus to preserve as much meniscus tissue as possible in the therapy of meniscus injuries. Thus, different techniques for the therapy of meniscus tears have been developed over time.

**Fig. 1:**
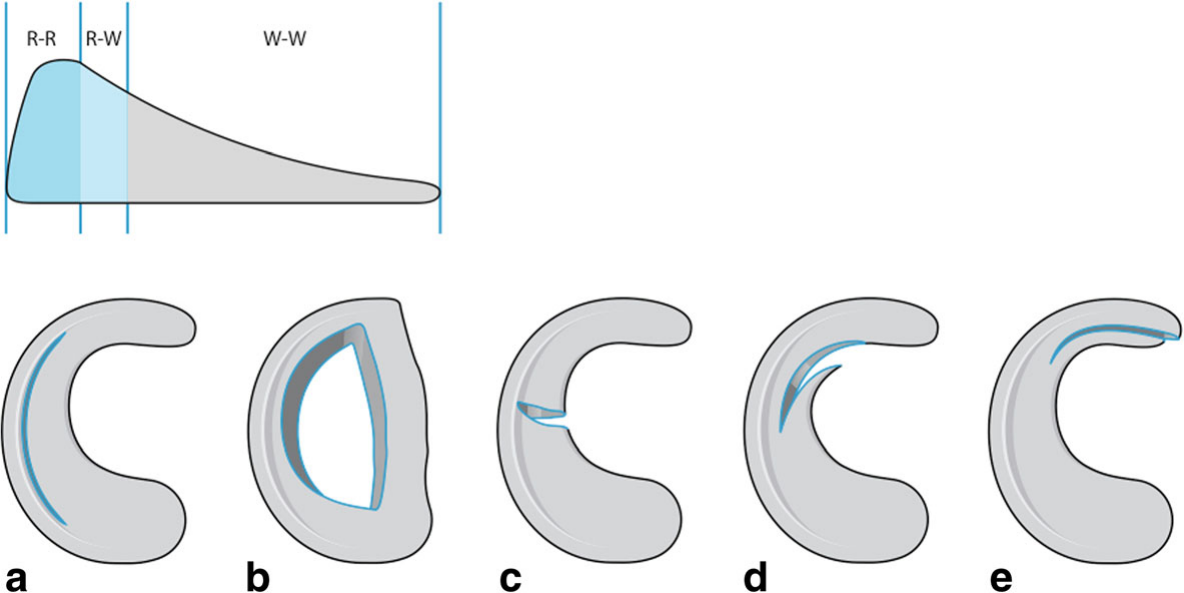
Schematic drawing: Mensical cross section, different tear types and their suturing potential. R-R: red-red-zone, R-W: red-white zone. W-W: white-white-zone: **a** longitudinal tear, **b** bucket handle tear, **c** radial tear, **d** flap lesion, **e** flap lesion with meniscal root participation. Different meniscal tear types: especially large bucket handle tears and radial tears that extend to the meniscal rim should be considered to be repaired

**Fig. 2:**
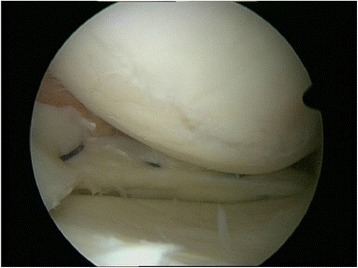
Meniscus suture. Forty two years old woman, medial meniscus left knee: performed meniscus suture in the red-white area in an early degenerative knee joint. To support endogenous meniscal regeneration by vascular supply a trephination of the meniscus basis was performed prior to the suture. (published in (Zellner & Angele, 2017))

Today, meniscus suturing can be seen as gold standard for the regenerative treatment of meniscus lesions (Figs. 1 and 2). Whereas initially this procedure was performed as an open procedure, up to suturing have to be differed: all-inside, outside-in, inside-out.

The vascularization and nutritional situation of the injured meniscus area as well as the type of meniscus tear are decisive of the success of a meniscus reconstruction.

While the inner 2/3 of the meniscus (“white-white”) is nourished by diffusion from the synovial fluid, the periphery in the so-called red-red zone has a vascular supply. Between the white-white zone and the vascularized portion a red-white transition zone is located.

Especially the outer third and, to a lesser extent, the red-white transition zone show a regenerative potential with good preconditions for a successful meniscus suturing (Arnoczky, 1999).

The question that arises is whether the patient has a benefit in the short or long term course by maintenance of meniscal substance, and whether, especially in contrast to meniscectomy, the reconstruction of the meniscus can prevent the development of an arthrosis.

### Success and failure of meniscus suturing is dependent on localization, time after trauma and genesis

The general advantage of the (partial) meniscectomy is usually described as a short-term significant improvement of the symptoms and fast rehabilitation. However, even the meniscus suturing is described to go along with good clinical results in more than 80% of the cases in the short-term outcome (Hulet et al., n.d.). In addition to that, analyzing the short term outcome of patients after partial meniscectomy and meniscus suturing Biedert found that both groups had comparable clinical and radiological results in the IKDC score and postoperative MRI (Biedert, 2000). Thus he concluded that the integrity and function of the meniscus is preferably preserved by meniscus suturing. So, especially in the vascularized red-red zone of the meniscus the endogenous regenerative potential has to be used. Further influencing factors such as sex of localization of the meniscus lesion (medial/lateral site) seemed to not affect the outcome after meniscus preserving therapy.

In addition, the meniscus also has a regenerative potential in the more critical transition zone (Fig. 1). For example, Barber-Westin showed in his review that the healing rate of meniscus suturing in the critical red-white zone was between 71% and 84%, depending on the study (Barber-Westin & Noyes, 2014).

An extended time from meniscus injury to therapy does not represent an absolute contraindication against a reconstructive treatment option. Espejo-Reina et al. report a success rate of 83% after refixation of bucket handle tears aged between 2 and 60 months (Espejo-Reina et al., 2014). Nevertheless, the healing rate of a meniscus suturing increases, the sooner it is performed. Meniscus lesions, which are treated by a reconstructive technique within 12 weeks, showed a better prognosis (Venkatachalam et al., 2001). These results correlate with the plastic deformation and roll-out phenomenon of older meniscal ruptures, which make a reduction and suturing more difficult. Thus, recent meniscus injuries should be treated promptly.

In a local patient population, 233 recent ACL ruptures with concomitant injuries of cartilage or meniscus being treated within one year after injury were analyzed. The concerned patients were divided into the ones being treated within the first 6 months after injury (86.3% of patients) and the ones being treated between month 7 to 12 (13.7%). The cartilage lesions did not show any differences in treatment at these time points. In contrast, it was found that meniscus injuries associated with ACL reconstruction could be better reconstructed if the surgery was performed in the course of the first 6 months after trauma (77.2% reconstructions of medial menisci within the first 6 months as opposed to 46.1% medial meniscus suturing in the months 7–12 after injury) (Krutsch et al., 2017). These results were confirmed by a further study. Thus meniscus sutures, which were performed within 3 months after trauma, show a higher rate of success (91%) than the treatments at a later date (58% success rate) (Venkatachalam et al., 2001).

Regarding the genesis of the injury, a study showed advantages for traumatically caused injuries unlike degenerative ones in relation to the healing potential (Venkatachalam et al., 2001).

In everyday clinical practice certain algorithms have evolved reflecting this situation. Younger patients with a recent meniscus lesion are more likely to be sutured, whereas older patients with degenerative meniscus lesions, which are an expression of a (starting) general joint wear, receive an arthroscopic partial meniscectomy. In the latter case, however, conservative therapy should also be primarily discussed and considered (Mezhov et al., 2014).

The current literature contains different studies about the failure rate after meniscus suturing. Johnson et al. report a secondary meniscectomy rate of 24% within 10 years after meniscus reconstruction (Johnson et al., 1999). Also Nepple et al. documented a meniscus suturing failure rate of 23%regarding an observation period of at least 5 years (Nepple et al., 2012). However, most of these long-term outcome studies/ meta-analysis refer to antique meniscus suturing techniques that were predominantly performed within an open procedure.

Regarding recent arthroscopically meniscus suture techniques, an further improvement of long-term outcomes and reduction of the failure rate is described. Lozano et al. reviewed the outcome after all-inside meniscus suturing and found a mean failure rate of 15% (Lozano et al., 2007).

Interestingly, the long-term analysis of Nepple et al. showed that the group of failed meniscus sutures were associated with a higher rate of radiological signs of osteoarthritis in the follow-up than the group of patients who had a successful meniscus suturing (Nepple et al., 2012). Based on this fact, it has to be assumed that the intact meniscus tissue has a protective effect on the surrounding cartilage, also regarding a future development of degenerative changes.

To positively influence the success rate of meniscus suturing a precise analysis of the joint status is required. Similar to the preoperative planning in the regenerative therapy of cartilage lesions the evaluation and therapy of additional present joint comorbidities, such as the alignment and joint stability, are a requirement for a successful meniscus reconstruction. Especially unstable joint situations increase the risk for meniscus suturing failures (DeHaven et al., 1995).

### How to improve the regenerative potential of the meniscus tissue

The aim of any meniscus therapy should be to preserve as much meniscus tissue as possible, such as performed by reconstructive techniques like the meniscus suture. The regenerative potential of the meniscus can be further supported by various measures.

**Fig. 3:**
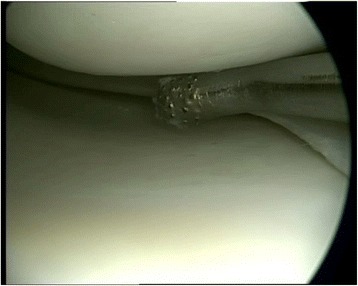
Preparation of the meniscus lesion. Refreshing of the margins of a meniscus tear by a rasp to induce a slight bleeding as an obligate procedure before meniscus suture to enhance meniscus regeneration (published in (Zellner & Angele, 2017))

So, the refreshing of the margins of meniscus tears is an obligate procedure before each meniscus suture (Fig. 3). Different techniques, such as the trephination of the meniscus margins by awls or K-wires as well as roughening of the defect sites by special meniscus tissue rasps, are available. In a comparative study, Zhang et al. analyzed the effect of such a refreshment of the meniscus defect site by trephination before meniscus suturing (Zhang & Arnold, 1996). They found a significantly lower failure rate of the meniscus sutures when, in addition to the suture, a trephination was performed before.

In addition to that, a beneficial joint milieu can positively influence the meniscus regeneration. Cannon et al. detected an increased healing rate of 93% in patients after meniscus suturing and simultaneous ACL replacement in comparison to a healing rate of 50% in patients, who had an isolated meniscus suture without simultaneous ACL replacement (Cannon Jr & Vittori, 1992). This fact has led to a marked increase of the number of meniscus sutures in combination with an ACL replacement in recent years. However, the positive effect presumable bases on the opening of the bone marrow space by drilling the femoral and tibial channels for the ACL replacement. Via these medullary canals mesenchymal stem cells as well as bioactive substances, which support the meniscus regeneration, may influence the joint milieu and arrive to the meniscus defect site. To imitate this effect, some authors also recommend a trephination of the notch before meniscus suturing to support the meniscus healing (Mordecai et al., 2014).

In the future biological augmentation of a meniscus suture, e.g. platelet rich plasma (PRP) or mesenchymal stem cells, could provide an advantage for the regeneration of the bradytrophic meniscus tissue in terms of extension of the indication and reduction of the treatment failure rate of meniscus suturing. Results of own, preclinical studies showed meniscus lesions in the avascular region for example could be healed by the use of mesenchymal stem cells (Zellner et al., 2013; Angele et al., 2008).

Another technique of aumenting mensical tears could be the use of fibrin as material filling the cleft before tightening the futures. The application of fibrin clot repair is intended to serve as a scaffold for repair and to stimulate a reparative process, especially in peripheral lesions (Taylor & Rodeo, 2013). First few results are promising (Taylor & Rodeo, 2013; Kamimura & Kimura, 2014; Jang et al., 2011).

Long-term data of the prevention of osteoarthritis by meniscus reconstruction are also missing for animal models. However the resection of meniscal tissue leads to cartilage lesions and degenerative changes of the knee after a very short time (Zellner et al., 2017).

### Treatment failure and complications

The question which arises when looking at current literature is what exactly has to be considered as a therapy failure. Therefore, a MRI follow up is not always meaningful. Pujol et al. analyzed MRI examinations of the knee joint 10 years after meniscus suturing and found a hyper intense signal in the treated meniscus in 87% of the cases. The authors concluded that MRI is not suitable for the analysis of the healing status of the meniscus after meniscus suturing (Pujol et al., 2013).

If a treatment failure is defined as a re-operation, there is a clear advantage for the partial meniscectomy. In a review Paxton et al. analyzed 95 studies regarding the outcome and re-operation rate after meniscus therapy (Paxton et al., 2011). For the period of 0 to 4 years after the first meniscus surgery they found a re-operation rate of 1.4% in the meniscectomy group as opposed to 16.5% in the meniscus suturing group. In the observation period longer than 10 years, a ratio of 3.9% for meniscectomy to 20.7% for meniscus suture was detected. However, the re-operation was defined as a further meniscus therapy. Whether and how many patients in which group had to be converted to for example an arthroplasty procedure remains unclear. Nevertheless, it is assured that the meniscus suture has a higher revision rate over time. This is a fact, which the patient has to be explicitly informed about before meniscus therapy.

On the other hand, the revision surgery has not necessarily to be classified as a complete failure of the meniscus suture. Pujol et al. showed that a partial reconstruction of the meniscus is also possible (Pujol et al., 2011). In 37 patients, the amount of meniscal substance resected during the revision was compared with the initial rupture. They found that in 52% approximately the same amount, but in 35% of the cases even less meniscus tissue had to be removed during the revision surgery. Regarding the fact that more meniscus tissue also means enhanced protection for the surrounding cartilage, this could also have a positive effect for the long-term outcome.

### Long-term results of meniscus suturing

The first description of a meniscus suturing technique was published by Annandale in 1885 (Di Matteo et al., 2013). Since then, the treatment options for the reconstructive therapy of meniscus lesions have been significantly advanced, especially by the development of arthroscopically techniques. Regarding studies and meta-analysis describing the long term outcome after meniscus reconstructive therapy, the technical development of the treatment options (open versus arthroscopically procedures) have to be considered.

Overall, current literature (Table 1) shows a significant positive effect of a meniscus tissue preserving therapy on the knee joint function in long-term. However, the question remains to what extend meniscus preserving techniques, such as meniscus suturing, are able to positively influence the development of degenerative changes within the knee joint.

**Table 1:** Overview of performed studies conferning long-term outcome after meniscus suturing

Author	Study design	Follow-up period	Results
Tengrootenhuysen et al. 2011 (Di Matteo et al., 2013)	Retrospective cohort study, *n* = 119	Follow up up to 5 y	Improved IKDC & Lysholm after meniscus suturing
Xu et al. 2015 (Tengrootenhuysen et al., 2011)	Meta analysis (*n* = 367), comparison of meniscus suturing vs. partial meniscectomy	Follow up up to 7 y	Improved IKDC & Lysholm as well as reduced loss of function in suturing group
Stein et al. 2010, (Xu & Zhao, 2015)	Cohort study (*n* = 81): comparison of meniscus suturing vs. partial meniscectomy	Follow up up to 9 y	Higher return to pre-injury activity level in suturing group
(Majewski et al., 2009)	Retrospective cohort study, *n* = 84	Follow up up to 10 y	rotational stability after successful suturing as high as in uninjured knees

**Fig. 4:**
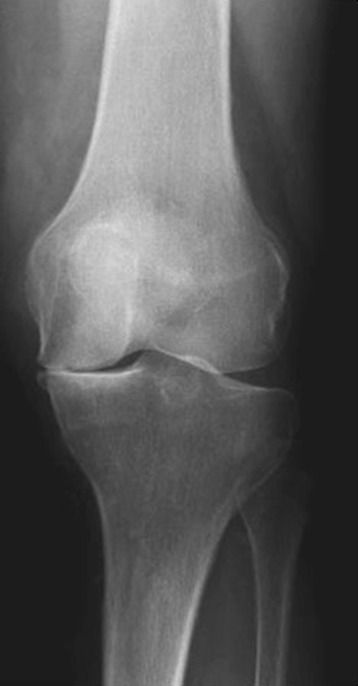
X-ray post subtotal meniscectomy. Forty two year old man: X-ray of a left knee joint 7 years after subtotal meniscectomy. It shows a lost joint line on the medial site and signs of osteoarthritis of the knee (published in (Zellner & Angele, 2017))

The integrity of the meniscus is of impact for the prevention of osteoarthritis, such as shown by (partial) meniscectomy. It usually goes along with a loss of symptoms and functional improvement in short-term (Mezhov et al., 2014). However, the long-term outcome after (partial) meniscectomy shows a trend to a degenerative effect. Englund and Lohmander described an association between the degenerative effect and the amount of lost meniscus tissue (Stein et al., 2010). Even if the partial meniscectomy does not show that extended destructive effect, osteoarthritic changes are also documented after a follow up of 16 years after partial meniscectomy (Englund & Lohmander, 2004). So, Papalia et al. defined the amount of resected meniscus tissue as a predictive factor for the development of osteoarthritis (Englund et al., 2003) (Fig. 4).

In a systematic review concerning the outcome after arthroscopically partial meniscectomy including a minimal follow-up of 8 years and a mean age of 36 years by Petty et al. satisfying results concerning the functional outcome (i.e. Lysholm scoring, Tegner scoring or IKDC scoring) were found (Petty & Lubowitz, 2011). Nevertheless, all included studies evaluating radiologically based signs of osteoarthritis in the index and contralateral site detected significantly enhanced signs of osteoarthritis in the group having a knee operation. Comparing the medial and lateral meniscus, especially partial meniscectomy of the lateral meniscus shows a negative influence on the development of degenerative changes (Papalia et al., 2011). In this context Lee et al. examining 49 patients after subtotal resection of the lateral meniscus and having lateral meniscus replacement throughout after a mean of 4.5 years. The authors observed a significant development of signs of osteoarthritis according to the Kellgren-Lawrence classification and a progressive loss of the joint line. Though, the process of progressive joint degeneration could have positively been influenced by meniscus replacement (Salata et al., 2010).

In case of untreated meniscus root tears, Steineman et al. 2017 (Steineman et al., 2017) demonstrated early OA changes in an animal model when leaving anterior meniscal root tears untreated for 8 weeks. Currently, no clinical studies are available which analyse the influence of repairing mensical root tears on progression of osteoathritis.

In contrast to the (partial) meniscectomy meniscus preserving techniques such as meniscus suturing show a cartilage protective effect in long-term. Noyes et al. evaluated the meniscal status of 33 patients having meniscus suture after a mean follow-up of 16.8 years by MRT scan. No degenerative changes in the operated compartment or differences concerning the status of degeneration in comparison to the healthy, contralateral site were found in patients after having successful meniscus suturing (Lee et al., 2016). Further studies described a progress of radiological signs of osteoarthritis after meniscus suturing in long-term, however these results showed just a mild progress of degenerative changes (Nepple et al., 2012). Johnson et al. compared the injured and contralateral knee joint 10 years after meniscus suturing on a radiological base (Johnson et al., 1999). While 8% of these patients developed osteoarthritic signs on the operated site, degenerative changes were also found in 3% of the contralateral, intact knee joints. Furthermore Tengrootenhuysen et al. analysed differences between patients after a successful meniscus suture and patients in whom the meniscus suture failed (Di Matteo et al., 2013). In 14% of the patients having a successful reconstruction of the meniscus signs of osteoarthritis were documented in X-ray. In contrast to that, in more than 80% of the patients with a failed meniscus preserving therapy signs of an osteoarthritis were seen.

Regarding the development of gonarthrosis, techniques preserving a functional intact meniscus tissue are also of advantage in comparison to partial meniscectomy. Stein et al. showed no progress of radiological signs of osteoarthrosis in 81% of the evaluated patients after almost 9 years after meniscus suturing, whereas a stop of a degenerative progress was only seen in 40% of the patients after partial meniscectomy (Xu & Zhao, 2015). Similar results were found by Paxton et al. (Paxton et al., 2011). While 78% of the patients had no progress of the osteoarthritic status according to the X-ray after having reconstruction of the meniscus, just in 64% of the patients, who had partial meniscectomy, no further development of gonarthrosis was detected. Especially in younger patients further studies showed also clear advantages of the meniscus preserving techniques in contrast to the partial meniscectomy concerning osteoarthrosis preventing qualities (Noyes et al., 2011).

## Conclusion

Meniscus preserving techniques to obtain a functional intact meniscus after meniscus injury in long-term are of great importance for the prevention of the development of osteoarthritis in the knee joint. Different significances and distributions of the studies are probably based on the heterogeneity of the evaluated patients, the different operation techniques and outcome analyses.

The authors of the manuscript recommend, based on the studies cited above, to try to perform meniscus preserving techniques, such as meniscus suturing, whenever achievable, and if a partial meniscetomy is necessary, to save as much meniscus tissue as possible.

Additionally, with suture treated patients always have to be informed about the failure rate, the extended period of rehabilitation and the higher rate of surgical revisions. However, further research projects, concerning for example augmented meniscus suture techniques, are necessary to reduce the failure rate and extend the indicational criterias for the meniscus preserving techniques.

## Abbreviations

ACL: Anterior cruciate ligament
IKDC: International knee documentation committee
MRI: Magnetic resonance imaging
PRP: Platelet rich plasma
